# Effects of diluting medium and holding time on sperm motility analysis by CASA in ram 

**Published:** 2014

**Authors:** Somayeh Mostafapor, Farhad Farrokhi Ardebili

**Affiliations:** *Department of Animal Science, Faculty of Agriculture, Urmia University, Urmia, Iran.*

**Keywords:** CASA, Ram, Semen diluters, Sperm motility

## Abstract

The aim of this study was to evaluate the effects of dilution rate and holding time on various motility parameters using computer-assisted sperm analysis (CASA). The semen samples were collected from three Ghezel rams. Samples were diluted in seminal plasma (SP), phosphate-buffered saline (PBS) containing 1% bovine serum albumin (BSA) and Bioexcell. The motility parameters that computed and recorded by CASA include curvilinear velocity (VCL), straight line velocity (VSL), average path velocity (VAP), straightness (STR), linearity (LIN), amplitude of lateral head displacement (ALH), and beat cross frequency (BCF). In all diluters, there was a decrease in the average of all three parameters of sperms movement velocity as the time passed, but density of this decrease was more intensive in SP. The average of ALH between diluters indicated a significant difference, as it was more in Bioexcell in comparison with the similar amount in SP and PBS. The average of LIN in the diluted sperms in Bioexcell was less than two other diluters in all three times. The motility parameters of the diluted sperms in Bioexcell and PBS indicated an important and considerable difference with the diluted sperms in SP. According to the gained results, the Bioexcell has greater ability in preserving motility of sperm in comparison with the other diluters but as SP is considered as physiological environment for sperm. It seems that the evaluation of the motility parameters in Bioexcell and PBS cannot be an accurate and comparable evaluation with SP.

## Introduction

Sperm motility is one of the most important effective factors in male animals' fertility.^1^ Therefore, the evaluation of the motility of sperm is one of the most important methods in the male animals' fertility. Numerous methods have been introduced for evaluation of semen quality (motility and morphology) in various breeds.^2^^-^^4^ In the mid-1980s, sperm analysis method by the use of computer of CASA as a proper substitute method for genuine calculation from sperms' morphological and motility features came on to the market.^5^ Computer assisted sperm analysis (CASA) allows the objective determination of the concentration of spermatozoa, a variety of motility parameters, and the assessment of morphological aberrations.^6^ However, each CASA system needs standardization and validation concerning semen preparation, calibration and technical settings to provide accurate and repeatable results.

Many authors have noted the need for standardization of procedures for semen handling to improve the reproducibility of results.^7^^-^^9^ Potential sources of error should be minimized, to allow comparison of data obtained by different systems and groups.^8^^,^^10^ Because of the high concentration of fresh ruminant semen, dilution is usually required for further analysis in a CASA. When the sperm concentration exceeds about 50 × 10^6^ sperm/ml it is necessary to dilute the semen containing concentrated sperm to obtain valid results with CASA. If the predilution for CASA is done accurately, the sperm concentration in the original semen sample can also be calculated accurately from CASA measurement,^9^ however, semen dilution may cause changes in motility parameters.

Another one of the common potential sources of variability in estimating the parameters for various semen characteristics measured by CASA is the time elapsed between the initial sampling and the analysis. The aims of this study were to evaluate effect of the dilution medium and holding time on various motility parameters of ram semen by CASA.

## Materials and Methods

**Semen Collection. **The semen samples were collected from three Ghezel rams using artificial vagina during breeding season. The rams were kept on the farm of Faculty of Agriculture, Urmia University. Two consecutive samples were taken from each ram with the interval of at least half an hour. The first samples used for recovery of seminal plasma (SP) and the second samples used for CASA. Seminal plasma was obtained by spinning of semen at 9000 rpm for 5 min, at 4 ˚C, in a micro-centrifuge (Model Universal 320R; Hettich Lab Company, Tuttlingen, Germany). The supernatant was centrifuged again, and SP was recovered.

**Evaluation of the semen. **Ejaculates having a sperm concentration of more than 2.8 × 10^9^ spermatozoa per mL, volume of more than 0.8 mL, at least 90% motility and dead sperm less than 5% were used for CASA. Semen collected from each ram were pooled after initial evaluation and used as single ejaculate. 

**Dilution of the semen. **Three media were used for dilution of semen: SP that prepared as described before, PBS with 1% bovine serum albumin (BSA) and Bioexcell (IMV Technologies, l’Aigle, France) as a commercial semen extender. Then 5 µL of pooled semen was added to 1 mL of each medium and then kept in water bath (37 ˚C) until motility evaluation. The sperm concentration in diluted semen was about 25 × 10^6^ per mL that provided sufficient sperm for CASA while minimizing errors due to sperm collisions. Following dilution, all semen samples divided to three subsamples were held in 1.5 mL micro tubes at water bath (37 ˚C) until motility evaluation. The sub-samples were used for CASA analysis after 0, 1, and 2 hr.

**Motility analysis. **Motility of diluted semen was assessed using a CASA system (Test Sperm 2.1; Videotest, St. Petersburg, Russia). This system included a phase contrast microscope (Model LX400; Labomed Inc., Culver City, USA) with a stage warmer, a sperm chamber with 10 µm deep (Sperm meter, Sperm Processor, Aurangabad, India), a CCD-camera (SDC-313B, Samsung Techwin Co., Gyeong, Korea). For each evaluation 5 µL of diluted sample was placed in the sperm chamber and up to 15 microscopic fields were analyzed to include at least 100 spermatozoa. Motion parameters shown by CASA software are: (1) average path velocity (VAP, µm sec^-1^):The average velocity of the smoothed cell path, (2) straight line velocity (VSL, µm sec^-1^): The average velocity measured in a straight line from the beginning to the end of track, (3) curvilinear velocity (VCL, µm sec^-1^): velocity of spermatozoa motion along individual tracks, (4) amplitude of lateral head displacement (ALH, µm): mean width of the head oscillation, (5) beat cross frequency (BCF, Hz): frequency of sperm head crossing the sperm average path in either direction, (6) straightness (STR, %): The departure of the cell path from a straight line and is defined as (VSL/VAP) ×100, (7) linearity (LIN, %). Degree of straightness of the cell track and defined as (VSL/VCL) ×100. Percentage of sperm motility assessed subjectively using the phase contrast microscope on same fields used for CASA. 

**Statistical analysis. **Data obtained were subjected to 3×3 factorial analysis in a completely randomized design (randomized complete block design) with six replication. Data were expressed as the mean ± standard error of mean (SEM). Analysis of variance was performed using the general linear model (GLM) procedure in SAS (Version 9.1, SAS Institute Inc., Carry, USA). The comparison of the means was done by Tukey’s test at a probability level of 5 %.

## Results

The percentage of sperm motility in 0, 1 and 2 hr after diluting in SP was 94%, 78% and 72% and in PBS 98%, 94% and 82% and in Bioexcell was 99%, 96% and 95%, respectively.

The effects of extender, holding time and the interaction extender × holding time were significant for VCL, VSL, VAP, STR and LIN (*p* < 0.05). Whereas in ALH the effects of extender and holding time and in BCF only the effects of extender was significant (*p *< 0.05). 

Data on sperm velocity parameters (VAP, VSL and VCL) of semen diluted in Bioexcell, BPS and SP after 3 holding times are shown in Tables 1, 2 and 3, respectively. The above mentioned parameters declined with increasing the holding time in all media (*p *< 0.05). The decrease in velocity parameters was more prominent in SP than in Bioexcell or PBS. Whereas, the motility pattern parameters (STR and LIN) were relatively uniform in diluted semen at holding times (Tables 1 and 2). The VAP and VSL were higher in semen diluted in PBS and lower in SP, (Tables 3 and 4). In Bioexcell this parameters were intermediate. The VCL was lower in sperm held for 0, 1 or 2 hr in SP, (Table 5). The straightness of sperm held for 0 or 1 hr was significantly higher in SP than PBS or Bioexcell, whereas nonsignificantly difference was observed between extenders after holding for 2 hr, (Table 4). The linearity of sperm trajectory was lower in Bioexcell than SP when held for 0 or 2 hr, (Table 1).

In sperms held for 2 hr, ALH was significantly lower than sperms held for 0 or 1 hr, (*p* < 0.05). The sperm that diluted in Bioexcell had high ALH than PBS and in both extender more than SP, (*p* < 0.05). Also, in BCF significant difference between SP and Bioexcell was observed, (*p* < 0.05).

**Table 1:** Mean (± SEM) straightness (%) of sperm diluted in different extenders during holding times (hr).

**Holding time**	**Extender**		
	**Seminal plasma**	**PBS**	**Bioexcell**
**0**	97.70 ± 0.26^A^	95.00 ± 0.38^B^	94.00 ± 0.63^B^^a^
**1**	98.23 ± 0.14^A^	96.00 ± 0.19^B^	95.90 ± 0.48^B^^b^
**2**	96.73 ± 0.48	96.40 ± 0.45	95.60 ± 0.07^a^^b^

ABC Different capital letters on the same row indicate a statistical difference between extenders (*p* < 0.05).

abc Different lower case letters on the same column indicate a statistical difference between holding times (*p* < 0.05).

**Table 2:** Mean (± SEM) linearity (%) of sperm diluted in different extenders during holding times (hr).

**Holding time**	**Extender**		
	**Seminal plasma**	**PBS**	**Bioexcell**
**0**	67.19 ± 1.10^A^	60.90 ± 1.93^B^^a^	55.90 ± 1.47^B^
**1**	62.74 ± 2.17^A^^B^	65.00 ± 1.00A^a^^b^	58.30 ± 0.80^B^
**2**	66.17 ± 0.97^A^	69.30 ± 1.19^A^^b^	53.40 ± 0.72^B^

ABC Different capital letters on the same row indicate a statistical difference between extenders (*p* < 0.05).

abc Different lower case letters on the same column indicate a statistical difference between holding times (*p* < 0.05).

**Table 3:** Mean (± SEM) average path velocity (μm sec^-1^) of sperm diluted in different extenders during holding times (hr).

**Holding time**	**Extender**		
	**Seminal plasma**	**PBS**	**Bioexcell**
**0**	97.70 ± 0.26^A^	95.00 ± 0.38^B^	94.00 ± 0.63^B^^a^
**1**	98.23 ± 0.14^A^	96.00 ± 0.19^B^	95.90 ± 0.48^B^^b^
**2**	96.73 ± 0.48	96.40 ± 0.45	95.60 ± 0.07^a^^b^

ABC Different capital letters on the same row indicate a statistical difference between extenders (*p* < 0.05).

abc Different lower case letters on the same column indicate a statistical difference between holding times (*p* < 0.05).

**Table 4:** Mean (± SEM) straight line velocity (μm sec^-1^) of sperm in diluted different extenders during holding times (hr).

**Holding time**	**Extender**		
	**Seminal plasma**	**PBS**	**Bioexcell**
**0**	93.63 ± 0.63^a^^C^	105.5 ± 1.41^a^^A^	100.10 ± 0.30^a^^B^
**1**	72.07 ± 1.09^b^^C^	101.3 ± 0.58^b^^A^	96.70 ± 0.88^a^^B^
**2**	60.74 ± 0.78^c^^C^	98.1± 0.67^b^^A^	87.20 ± 0.78^b^^B^

ABC Different capital letters on the same row indicate a statistical difference between extenders (*p* < 0.05).

abc Different lower case letters on the same column indicate a statistical difference between holding times (*p* < 0.05).

**Table 5:** Mean (± SEM) curvilinear path velocity (μm sec^-1^) of sperm diluted in different extenders during holding times (hr).

**Holding time**	**Extender**		
	**Seminal plasma**	**PBS**	**Bioexcell**
**0**	142.36 ± 2.22^a^^B^	178.80 ± 4.23^a^^A^	183.80 ± 4.18^a^^A^
**1**	116.99 ± 3.00^b^^B^	160.20 ± 2.86^b^^A^	170.30 ± 1.81^a^^b^^A^
**2**	93.59 ± 1.91^c^^C^	146.50 ± 3.36^b^^B^	166.60 ± 2.64^b^^A^

ABC Different capital letters on the same row indicate a statistical difference between extenders (*p* < 0.05).

abc Different lower case letters on the same column indicate a statistical difference between holding times (*p* < 0.05).

## Discussion

Until recent years, assessment of sperm quality had been based on subjective evaluation of parameters, such as mass and individual motility. When subjective optical microscopic evaluation was used in human being and animals, variations of 30 to 60% have been reported in the estimation of motility parameters of the same ejaculates.^5^ Computer assisted sperm analysis has allowed an objective and accurate approach for sperm motility assessment.^11^ Recognition of effective factors in sperm analysis by CASA and introduction of necessary standard have critical value increasing accuracy and comparability of study results. In animals with high sperm concentrations, diluent type is one of the most effective factors on variability of the results by CASA. Several authors have discussed the need to reduce sperm concentration to less than 50 × 10^6^ sperm per mL.^12^^-^14 Homologous SP is frequently used to dilute specimens.^12^^,^^13^^,^^15^ However, SP varies, it can affect measurements, and it may not be available when evaluating semen other than fresh specimens. Crittenden and Handelsman recommended changing software instead of dilution to minimize bias in CASA analysis of fresh and cryopreserved semen.^16^

The results of the present study showed that the diluent medium influenced the sperm motility parameters in semen evaluation by CASA. In most studies the effect of diluting medium on sperm motility parameters were reported.^17^^-^21 Farrell *et al*. reported that the effects of various diluting medium on sperm motility parameters in rabbit were noticeable but in human sperm, motion characteristics in the three diluents were more similar than were in those of rabbit sperm.^18^ Tardif *et al*. used 3 diluters Tyrode’s albumin lactate pyruvate (TALP), Cornell University extender (CUE) and egg yolk-glycerol-tris extender (EYGT) and reported that a significant difference in the evaluated motility parameters by CASA.^20^ Schafer-Sami *et al*. evaluated dog sperm motility patterns by CASA and used 4 diluting mediums and reported that the effects of various diluting medium on sperm motility parameters were noticeable.^21^

The three media had been in use in our laboratory for diluting sperm. The first extender was SP, a sperm physiological medium, the second extender was Bioexcell which is commercially available for bull semen and the third extender was the PBS with 1% bovine serum albumin.

Our results showed that the velocity values (VCL, VSL and VAP) of the diluted sperm in SP were significantly less than in Bioexcell and PBS. Although there was a significant difference in average amount of VCL and VAP between Bioexcell and PBS, the observed difference was not considerable. Low velocity of sperms in the SP can be as a result of high viscosity and its physical features. In a study in goat, Cox *et al*. used SP and Sperm Analysis Medium (SAM) as diluting mediums.^17^ They reported that the velocity parameters of sperms suspended in SP were considerably less than SAM medium. Robayo *et al*. used the same diluting medium on ram, and observed that the motility parameters of the diluted sperms in SP were considerably less than the diluted in sperm analysis medium (SAM).^19^ In our study values of ALH and BCF between three diluents showed a significant difference while the maximum amount was in Bioexcell and the minimum amount was in SP. In other words, the amplitude ALH, in Bioexcell was more than in SP and PBS. As it is observed in Tables 3, 4 and 5 the VCL in Bioexcell is more than PBS but the VAP and VSL are less than PBS. It is because of high amount of ALH of the sperms that diluted in Bioexcell. The STR had a slight difference between the diluents and in all cases it was over 94%, however, LIN in the sperms suspended in Bioexcell was less than two other diluters in all 3 hr of study, (Tables 1 and 2). It is because of high VCL in the diluted sperms in Bioexcell. In evaluation of the motility sperm parameter in each diluter during study time (0, 1 and 2 hr after dilution) decrease in velocity parameters of the diluted sperms in SP was more intense than the two other diluters. In other words, the capability of Bioexcell and PBS diluters in keeping motility sperm parameters were more than plasma (Tables 3, 4 and 5), however, changes of these parameters in Bioexcell and PBS were less and almost alike. Nevertheless the decrease in the motility of sperms in Bioexcell as the time passed (99%, 96% and 94%, respectively, in 0, 1 and 2 hr) was considerably less than those diluted in PBS (98%, 94% and 82%, respectively) and SP (94%, 78% and 72%). 

The effects of holding time on motility parameters of sperms in this study were different from the results in bull semen.^9^ There was little difference in most sperm characteristics associated with the way the bull semen was held for 2 hr without dilution or diluted in TALP. This different effect of holding time on motility parameters may be result of different extender (i.e. TALP) used for dilution. 

In conclusion, on the basis of this study the extender can affect the motility parameters of sperm. The velocity parameters of sperm (VCL, VCL and VAP) and lateral head displacement were high in semen diluted in Bioexcell and PBS than SP, however, sperms in SP have more linear trajectory than in Bioexcell or PBS. Another finding of this study was that Bioexcell can preserve motility of sperm better at holding time than PBS or SP. 

## References

[1] Davis RO, Katz DF (1992). Standardization and comparability of CASA instruments. J Androl.

[2] Bratton RW, Foote RH, Henderson CR (1956). The relative usefulness of combinations of laboratory tests for predicting the fertility of bovine semen. J Dairy Sci.

[3] Hafs HD, Hoyt RS, Bratton RW (1958). Effects of daily ejaculations on sperm output, fertility, and libido of dairy bulls. J. Dairy Sci.

[4] O’Conner MT, Amann RP, Saacke RG (1981). Comparison of computer evaluations of spermatozoa motility with standard laboratory tests and their use for predicting fertility. J Anim sci.

[5] Verstegen J, Iguer-Ouada M, Onclin K (2002). Computer assisted semen analyzers in andrology research and veterinary practice. Theriogenology.

[6] Thurston LM, Watson PF, Holt WV (1999). Sources of variation in the morphological characteristics of sperm sub-populations assessed objectively by a novel automated sperm morphology analysis system. J Reprod Fertil.

[7] Krause’ W, Viethen G (1999). Quality assessment of computer- assisted semen analysis (CASA) in the andrology laboratory. Andrologia.

[8] Kraemer M, Fillion C, Martin-Pont B (1998). Factors influencing human sperm kinematic measurements by the Cell track computer-assisted sperm analysis systems. Hum Reprod.

[9] Farrell PB, Trouern-Trend V, Foote RH (1995). Repeatability of measurements on human, rabbit, and bull sperm by computer-assisted sperm analysis when comparing individual fields and means of 12 fields. Fertil Steril.

[10] Smith SC, England GC (2001). Effect of technical settings and semen handling upon motility characteristics of dog spermatozoa measured using computer-aided sperm analysis. J Reprod Fertil Suppl.

[11] Mortimer ST (2000). CASA - Practical aspects. J Androl.

[12] Vantman D, Koukoulis G, Dennison L (1988). Computer-assisted semen analysis: evaluation of method and assessment of the influence of sperm concentration on linear velocity determination. Fertil Steril.

[13] Schrader SM, Chapin RE, Clegg ED (1992). Laboratory methods for assessing human semen in epidemiologic studies: a consensus report. Reprod Toxicol.

[14] Davis RO, Katz DF (1993). Operational standards for CASA instruments. JAndrol.

[15] Farrell PB, Foote RH, Simkin ME (l993). Relationship of semen quality, number of sperm inseminated and fertility in rabbits. J Androl.

[16] Crittenden JA, Handelsman DJ (1995). Density adjustment of software settings minimizes bias in automated sperm motility estimation. Hum Reprod.

[17] Cox JF, Alfaro V, Montenegro H (2006). Computer-assisted analysis of sperm motion in goats and its relationship with sperm migration in cervical mucus. Theriogenology.

[18] Farrell PB, Foote RH, Mc Ardle MM (1996). Media and dilution procedures tested to minimize handling effects on human, rabbit, and bull sperm for computer-assisted sperm analysis (CASA). J Androl.

[19] Robayo I, Montenegro V, Valdes C (2008). CASA assessment of kinematic parameters of ram spermatozoa and their relationship to migration efficiency in ruminant cervical mucus. Reorod Dom Anim.

[20] Tardif AL, Farrel PB, Trouern-Trend (1997). Computer-assisted sperm analysis for assessing initial semen quality and changes during storage at 5˚C. J Dairy Sci.

[21] Schafer- Sami S, Aurich CH (2007). Use of new computer-assisted sperm analyzer for the assessment of motility and viability of dog spermatozoa and evaluation of four different semen extenders for predilution. Anim Reprod Sci.

